# Voclosporin: Unique Chemistry, Pharmacology and Toxicity Profile, and Possible Options for Implementation into the Management of Lupus Nephritis

**DOI:** 10.3390/cells12202440

**Published:** 2023-10-11

**Authors:** Ajinath Kale, Vishwadeep Shelke, Yutian Lei, Anil Bhanudas Gaikwad, Hans-Joachim Anders

**Affiliations:** 1 Department of Pharmacy, Birla Institute of Technology and Science Pilani, Pilani Campus, Pilani 333031, Rajasthan, India; ajinathk3@gmail.com (A.K.); vishwadeepshelke@gmail.com (V.S.); anil.gaikwad@pilani.bits-pilani.ac.in (A.B.G.); 2 Division of Diabetology, Department of Internal Medicine IV, Hospital of the Ludwig Maximilians University Munich, 333031 Munich, Germany; yutian.lei@med.uni-muenchen.de; 3 Division of Nephrology, Department of Internal Medicine IV, Hospital of the Ludwig Maximilians University Munich, 80336 Munich, Germany

**Keywords:** calcineurin, autoimmunity, inflammation, proteinuria, podocytes, toxicity

## Abstract

Calcineurin inhibitors (CNI) can suppress allo- and autoimmunity by suppressing T cell function but also have anti-proteinuric effects by stabilizing the cellular components of the kidney’s filtration barrier. Therefore, CNI are used in autoimmune kidney diseases with proteinuria. However, the traditional CNI, cyclosporine A and tacrolimus, have a narrow therapeutic range, need monitoring of drug levels, and their use is associated with nephrotoxicity and metabolic alterations. Voclosporin (VOC), a novel CNI, no longer requires drug level monitoring and seems to lack these adverse effects, although hypertension and drug–drug interactions still occur. VOC demonstrated efficacy superior to standard-of-care in controlling active lupus nephritis in the phase 2 AURA-LV and the phase 3 AURORA-1 trials and was approved for the treatment of active lupus nephritis. However, how to implement VOC into the current and changing treatment landscape of lupus nephritis is still debated. Here, we review the unique chemistry, pharmacology, and toxicity profile of VOC, summarize the efficacy and safety data from the AURA-LV and AURORA-1 trials, and discuss the following four possible options to implement VOC into the management of lupus nephritis, namely regarding B cell-targeting therapy with belimumab (BEL). These include: 1. patient stratification to either VOC or BEL, 2. VOC/BEL combination therapy, 3. VOC-BEL sequential therapy, or 4. alternative options for the rapid antiproteinuric effect of VOC.

## 1. Introduction

Calcineurin inhibitors (CNI) are a class of immunosuppressive drugs used to suppress adaptive immunity during solid organ transplantation or in autoimmune disease [1,2]. CNI are particularly attractive for the treatment of proteinuric kidney diseases as unlike other immunosuppressants, CNI has specific antiproteinuric effects [3]. Indeed, autoimmune forms of glomerulonephritis can specifically benefit from this dual mechanism of action but CNI are also used in podocytopathies of unknown causes [4]. However, nephrotoxicity limits the long-term use of CNI [5].

Cyclosporin A (CsA) was the first CNI in use but has a narrow therapeutic range and a clinically relevant dose-dependent and dose-independent toxicity profile [6]. This prompted the search for other CNI with better toxicity profiles. Tacrolimus (TAC) was the next CNI approved for the treatment of allo- and autoimmunity [7]. Tacrolimus still has a narrow therapeutic range but comes with a partially different toxicity profile, which allows diversification of CNI’s use in patients with specific risk factors. However, CsA and TAC both require monitoring of drug levels and are subject to numerous drug interferences due to their specific mode of metabolism via cytochrome P450 [8]. VOC, a more recently developed CNI, no longer requires monitoring of drug levels and has a different toxicity profile [9]. Currently, VOC has only been tested in the clinical contexts of kidney transplantation and lupus nephritis. Its potent antiproteinuric effect makes it a perfect fit for this indication as the trial endpoint heavily relies on the proteinuria response, which is more difficult to achieve for immunosuppressant agents that do not directly modulate the glomerular filtration barrier. Here we review the molecular biology of CN as a target treatment, the pharmacology and toxicity of available CNI, and the potential use of VOC in the context of its latest approval for the treatment of active lupus nephritis (LN) [10].

## 2. Calcineurin as a Molecular Drug Target

**Figure 1:**
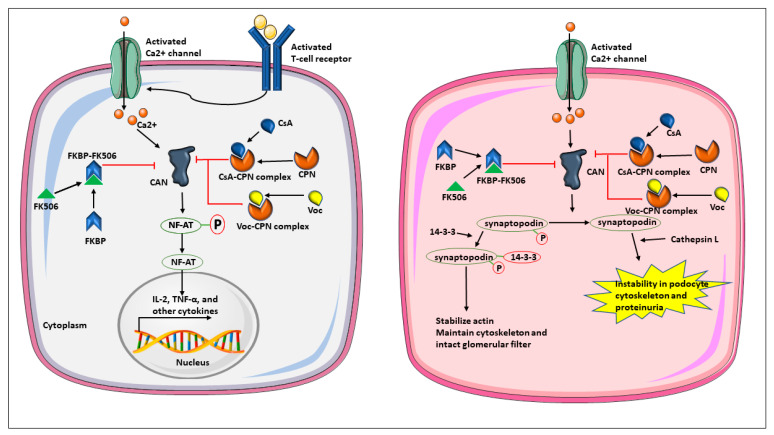
Roles of calcineurin in T lymphocytes (grey) and podocytes (pink). Phosphatase calcineurin (CN) is a Ca++ sensitive enzyme in the nuclear factor of activated T cell (NF-AT) signaling pathways downstream of the T cell receptor activated by foreign, auto-, or alloantigens. Cyclosporin A (CsA), Tacrolimus (FK506) and Voclosporin (Voc) all block the phosphatase activity of CN, albeit in different ways. In podocytes, CN dephosphorylates synaptopodin, a protein involved in the stability of the actin cytoskeleton. CPN = cyclophilin; FK506 = tacrolimus; FKBP = FK506-binding protein; CsA = cyclosporine A; Voc = voclosporin; IL = interleukin; TNF = tumor necrosis factor; NF-AT = nuclear factor of activated T cells.

CN is a calmodulin/calcium-activated serine-threonine phosphatase that regulates calcium signaling and immune and inflammatory processes. CN is a heterodimer composed of two subunits: the catalytic A subunit and the regulatory B subunit [11]. Catalytic subunit A shares the major portion and is responsible for calmodulin binding and associated downstream signaling. However, the regulatory subunit B has four calcium binding sites sensitive to calcium signaling. The calcium and calmodulin-mediated activation of calcineurin lead to the dephosphorylation of substrates involved in several critical cellular processes (Figure 1). Those substrates are not limited to receptors, channels, and proteins associated with microtubules and mitochondria, and, more importantly, transcription factors [12]. The most commonly studied transcription factors are nuclear factors of activated T-cells (NFAT 1-5), nuclear factor kappa-light-chain-enhancer of activated B cells (NFκB), the forkhead transcription factors, and transcription factor EB. These factors drive the release of pro-inflammatory cytokines as interleukins, interferons, CD40 ligand, tumor necrosis factors, and T-cell receptor (TCR) signaling involved in the activation of T lymphocytes responding to foreign antigens, autoantigens, or alloantigens [13]. Interleukins/cytokines further activate downstream signaling via phosphatidyl-inositol-3 kinase, and the mammalian target of rapamycin, which all regulate the activation, proliferation, and differentiation of T cells (Figure 1) [14].

Moreover, CN is also involved in the glomerular barrier function in kidney podocytes (Figure 1). Podocytes are epithelial cells in the kidney glomeruli with an essential role in maintaining integrity and function of the glomerular filtration filter [15]. Specifically, the interdigitating podocyte foot processes, connected by proteins of the filtration slit in a zipper-like structure to form a unique intercellular junction called a ’slit diaphragm’ that is essential for the kidney filter [16]. Any focal or diffuse injury to podocytes and their foot processes induces serum protein leakage into the urine, i.e., proteinuria [17]. Numerous proteins are involved in connecting the actin cytoskeleton of podocytes with the proteins in the slit diaphragm, e.g., synaptopodin [16]. Interestingly, CN can dephosphorylate podocyte synaptopodin, which destabilizes the actin cytoskeleton, the podocyte secondary foot processes, and the filtration slit [18]. Thus, CN-mediated dephosphorylation of synaptopodin contributes to proteinuria, podocyte loss, and glomerulosclerosis (Figure 1). Vice versa, CNI reverse these processes, and thus have antiproteinuric and renoprotective effects [18,19,20]. Together, CN is a molecular target in allo- and autoimmune and inflammatory diseases including SLE, LN, and other proteinuric kidney diseases.

## 3. Chemistry of the Calcineurin Inhibitors

### 3.1. Cyclosporin

CsA is a cyclic undecapeptide (C_62_H_111_N_11_O_12_) with a molecular weight of 1202 Daltons, it is neutral, hydrophobic, and possesses eleven amino acid residues from which seven amino acids are N-methylated, and the remainder are non-methylated [21,22]. During binding to cyclophilin, one N-methylated amino acid (Mle-3) bond of CsA remains in the trans-orientation [23]. Due to N-methylated amino acids, there are fewer chances of intramolecular hydrogen bonds, while the carbon chain of amino acid in position 1 (3-hydroxy-4-methyl-2-methylamino-6-octanoic acid, abbreviated as MeBmt) is essential for the bioactivity of CsA. The immunosuppressive effect significantly declines upon removal of the non-polar part of the side chain [24]. Interestingly, the modifications at amino acid 2 with some alkyl chains, improve the immunosuppressive activity of CsA [23]. All 11 amino acids show an S-configuration of natural L-amino acids except for D-alanine in the eighth position (R-configuration) and N-methyl-glycine in the third position. The hydrolysis of CsA yields a cyclic derivative of MeBmt, while an acid treatment of CsA provides iso-cyclosporine. X-ray crystallography and nuclear magnetic resonance measurements confirm that CsA in crystal and soluble form mainly differ in their respective confirmations due to the orientation of the carbon chain of the MeBmt amino acid [25]. However, several analogs were generated to improve the potency and efficacy of CsA.

### 3.2. Tacrolimus

TAC (also refered to as Fujimycin, tsukubaenolide, or FK506) is a member of the L-pipecolic acid-derived macrolides class and has at least one deoxy sugar connected to a large macrocyclic 14-16-membered ring as part of its 23-membered lactone ring [26]. The molecular formula of TAC is C_44_H_69_NO_12_ with a molecular weight of 803.5 Daltons and is generally found in a white to slightly off-white crystalline form. TAC is poorly soluble in water and has a LogP value of 3.3. However, in aqueous solutions, TAC undergoes cis-trans-isomerization and exists in three different forms, mainly due to changes at the C-12 position [27]. The limited and restricted rotation of the two amide bonds creates two conformational rotamers of TAC. Importantly, tautomeric structural modifications can be produced due to three adjacent carbonyl groups in the TAC ring, resulting in different TAC analogs [28]. The pipecolic acid moiety, free hydroxy groups, and tricarbonyl groups are essential for functional activity. The 2-propynyl group presented at the C-8 position is important for the interaction with the FK-binding protein (FK-BP) and calcineurin inhibition. Any modification at the C-8 position would result in instability in the FK-BP-TAC complex [29]. Moreover, the 2-propynyl group at C-8 replaced with -CH2-CH3 or -CH2-CH2=CH3 yields ascomycin and TAC 8-propyl analog, which further complicates the purification of TAC [27].

### 3.3. Voclosporin or ISA247

VOC (C_63_H_111_N_11_O_12_) is a cyclic undecapeptide like CsA but with a molecular weight of 1214.6 Daltons. It forms a heterodimeric complex with cyclophilin A that binds to CN [30]. To create VOC, a single carbon extension was placed at the amino acid 1 position of CsA. VOC is available in cis- and trans-isomers, from which the trans-isomer is considered more potent. These two isomers differ in the orientation of one functional group [31,32]. Similar to CsA, modification at the amino acid 1 position with single carbon results in a more prominent binding of the cyclophilin-VOC complex to the ‘latch-region’ of CN thus increasing the potency of VOC [30]. More importantly, the amino acid 1 position is essential for VOC metabolism. Therefore, any modifications at this position result in shifting the metabolism site from the amino acid 1 to the amino acid 9 position, producing less potent metabolite [33]. On the other hand, CsA undergoes a tremendous metabolism and yields highly nephrotoxic metabolites, which limit the use of CsA in kidney disease patients [34,35]. Therefore, the structural modifications at amino acid 1 renders VOC more safe and more potent compared to CsA [35].

## 4. Pharmacology of the Calcineurin Inhibitors

### 4.1. Cyclosporine A (Sandimmune^®^, Neoral^®^)

Since 1980, CsA has been widely used in autoimmune and inflammatory disorders and organ transplant recipients [36]. However, it has a narrow therapeutic range and can cause serious side effects which limit its chronic use or its use as a first-line therapy in many settings (Table 1) [36].

Pharmacokinetic: CsA is available in liquid (oral or intravenous solution) and solid dosage forms (capsules). Close therapeutic drug monitoring of CsA is recommended [37]. CsA is a lipophilic peptide and shows a variable pharmacokinetic profile. Oral absorption of CsA is low which further decrease with the presence of food. Upon oral administration, it can achieve bioavailability in a wide range [38]. CsA is extensively distributed in peripheral tissues, blood erythrocytes, and plasma. It binds with albumin and lipoproteins in the plasma [38]. The volume of the distribution of CsA lies between 3–5 L/kg and the reported half-life varies from 5–27 h. The liver and intestines are the major sites wherein CYP3A1,2,4,5,9, and CYP3C are the common enzymes responsible for CsA metabolism [39]. AM1, AM1c, AM9, AM1c9, and AM4N are the CsA metabolites in which AM19 is the major contributor to CsA-associated nephrotoxicity [6]. The underlying mechanisms of CsA-induced nephrotoxicity are not entirely clear; however, the p38, JNK, ERK, and MAPK subfamilies signaling are involved in CsA nephrotoxicity [40]. CsA is mainly eliminated via biliary excretion and feces (~70%), and only 6–15% via the urine [41].

Contraindications: CsA shows significant interactions with certain medications such as amphotericin B, neomycin, atorvastatin, cidofovir, elbasvir/grazoprevir, flibanserin, lomitapide, mifepristone, tacrolimus, etc. The CYP3A4 inhibitors like macrolides, verapamil, amiodarone, colchicine, oral contraceptives, and azole antifungals are known to increase the plasma concentrations of CsA, TAC, and VOC when used concomitantly. Moreover, CNI should not be given alongside CYP3A4 inducers which can decrease the plasma concentrations of these drugs to subtherapeutic levels, e.g., carbamazepine, orlistat, phenytoin, etc. Thus, close monitoring and/or dose adjustments are often required when calcineurin inhibitors are used in combination with these drugs.

Adverse effects: Arterial hypertension, dyslipidemia, hirsutism, hyperuricemia, hyperkalemia, and nephrotoxicity are more pronounced with CsA compared to TAC and VOC (Table 2). CsA increases the low-density lipid, triglycerides, apolipoprotein B, and lipoprotein (a) levels, and thus decreases the transportation of cholesterol to the intestines. CsA also reduces the process of lipolysis and levels of high-density lipids. Other common adverse effects include gynecomastia, arrhythmia, diabetes, a decrease in eGFR and creatinine clearance, convulsions, bleeding gums, gingical hyperplasia, hypertrichosis, and gastrointestinal upset.

### 4.2. Tacrolimus (Astagraf XL, Envarsus XR, Prograf, and Protopic)

TAC is a macrolide, one of the commonly prescribed immunosuppressants in combination with other drugs to prevent solid organ transplant rejection (heart, liver, lung, and kidney) [42]. Indeed, it is now used as a standard first-line therapy in kidney transplant recipients. TAC is also approved to treat skin disorders such as atopic dermatitis/eczema [43]. However, TAC has a narrow therapeutic window, its metabolism is disturbed by various factors, and hence requires therapeutic drug monitoring [8,44]. It also shows complex pharmacokinetics, pharmacodynamics, and pharmacogenetics among organ transplant recipients which limits its clinical applications [45,46].

Pharmacokinetics: TAC is formulated in oral, intravenous, and topical dosage forms (Table 1). The oral dosage form is designed in different release patterns: immediate, slow, and extended release [47]. It combines with one or more therapies such as monoclonal antibody-basiliximab, or drugs, such as sirolimus, azathioprine, mycophenolate mofetil, and steroids [48,49,50]. TAC is metabolized by CYP3A4, CYP3A5, and P-glycoprotein (PGP)/ABCB1 into the different metabolites, among which the main and major metabolite is 13-O-dimethyl-tacrolimus [45]. The half-life ranges from 4 to 41 h, and the approximate volume of distribution is 30 L/kg [45]. It is mainly excreted by the biliary route (~95%) and 2–3% is unchanged in the urine.

Contraindications: Antifungal agents and multiple other drugs metabolized by CYP450 enzymes, including polyoxyl 60 hydrogenated castor oil (HCO-60), and their derivatives require close drug level monitoring and/or dose adjustment to avoid TAC toxicity [51].

Adverse effects: Post-transplant diabetes mellitus, neurotoxicity, GIT upset, and alopecia are more pronounced with TAC when compared to CsA and/or VOC (Table 2). It causes diabetes by stimulating glucolipotoxicity in β cells and thus leads to decreasing insulin secretion. Nephrotoxicity, hypertension, dyslipidemia, angina pectoris, cardiac arrhythmias, hyperkalemia, urinary tract infections, cosmetic, and electrolyte disturbances are other common adverse events associated with TAC [52].

### 4.3. Voclosporin (Lupkynis™)

Voclosporin is a potent cyclosporine derivative, safer, more efficacious, and tolerable than other calcineurin inhibitors [53]. VOC received clinical approval from the USFDA to be used in combination with MMF and corticosteroids for treating LN [10]. However, against psoriasis, it is inferior to CsA in terms of efficacy.

Pharmacokinetics: VOC is available only in oral solid dosage form. For treating LN, the starting dose of VOC is 23.7 mg twice a day, with 8 or 12 h intervals (Table 1) [10]. VOC is recommended to be consumed on an empty stomach for its maximum absorption and optimum bioavailability. The Tmax lies between 1 and 4 h and Cmax is estimated at 955.5 ng/mL (https://www.accessdata.fda.gov/drugsatfda_docs/label/2021/213716s000lbl.pdf, Accessed on 10 January 2023). Moreover, 2154 L/kg is the apparent volume of distribution of VOC. Orally administered VOC is extensively metabolized by CYP 450 (3A4/5) in the liver, hence drugs with CYP450 induction or inhibition properties are not recommended to be administered concomitantly (https://www.accessdata.fda.gov/drugsatfda_docs/label/2021/213716s000lbl.pdf, Accessed on 10 January 2023). The half-life of VOC ranges from 25 to 36 h, the mean apparent steady-state clearance is 63.6 L/h, and it is excreted in feces (~88%) and urine (~2%) [10,31]. It is advised to be used in patients with a baseline eGFR of >45 mL/min/1.73 m^2^ wherein continuous eGFR monitoring is required. Moreover, it should be avoided in patients with eGFR of ≤45 mL/min/1.73 m^2^ or a baseline blood pressure of >165/105 mmHg and mild-to-moderate hepatic impairment.

Compared to other CNI, VOC has linear and predictable pharmacokinetic profiles [54]. Unlike CsA and TAC, it does not require therapeutic drug monitoring and thus a fixed dose can be prescribed. In addition, when CsA and MMF are combined, the enterohepatic circulation of MMF is inhibited, and plasma concentrations of mycophenolic acid (MPA) will be lower. Voclosporin does not affect the enterohepatic circulation of MMF, leading to higher MPA exposure and potentially better efficacy compared to an equivalent MMF dose combined with CsA [55]. In kidney transplant recipients and plaque psoriasis patients, VOC showed a strong correlation between dose, its systemic concentration, CN inhibition, efficacy, and the threat of adverse events [56,57].

Contraindications: CYP3A4 induces or inhibits P-gp and OATP1B1 substrates (statins), phenylalanine, flunisolide, bortezomib, and cladribine in patients with renal and hepatic impairments. Close monitoring as well as dosage adjustment are recommended when VOC is used along with such drugs.

Adverse effects: Overall, the safety profile of VOC is good when compared to CsA and TAC (Table 2). VOC has not been reported to produce the other CNI related serious adverse effects such as diabetes, gum hyperplasia, dyslipidemia, arrhythmia, and disturbance in electrolyte balance and metabolic profile. Other common but non-serious adverse effects, including increased blood pressure, diarrhea, headaches, anemia, coughs, UTIs, upper abdominal pain, dyspepsia, alopecia, abdominal pain, mouth ulceration, nausea, tremors, and decreased appetite, were occasionally observed. The most common adverse effect from VOC was a transient drop in eGFR.

## 5. Experience with Calcineurin Inhibitors in Autoimmune Glomerulonephritis and Podocytopathies

The current KDIGO guidelines recommend the use of CNI as a second line treatment in a number of autoimmune glomerular diseases, namely for better control of proteinuria [58]. Especially in podocytopathies presenting as steroid-dependent nephrotic syndrome, CNI are used as steroid-sparing agents [17,58]. In cases of steroid-resistant nephrotic syndrome, CNI are in use while awaiting the results of genetic testing [4]. In membranous nephropathy, a glomerulonephritis with an autoimmune podocyte injury [59,60], the results of the MENTOR trial have reduced the enthusiasm for the use of CsA because B cell depletion with rituximab was equally effective but, unlike CsA, did not show nephrotoxicity [61]. TAC in combination with rituximab was better tolerated but was inferior to cyclophosphamide in this context [62]. In contrast, studies from China convincingly demonstrate the superiority of a combination of MMF and TAC to reach traditional trial endpoints in LN that heavily depend on the proteinuria response [63,64]. Confirmation from other world regions remained pending due to the lack of sponsor interest to invest into trials with “old” drugs. This changed with the introduction of VOC.

### 5.1. Preclinical Data of Voclosporin and the Rationale to Develop Voclosporin for Lupus Nephritis

VOC can attenuate the severity of disease in rodent models of autoimmune uveitis and corneal inflammation [65,66]. However, experimental data on animal models of autoimmune glomerulonephritis, LN, or other proteinuric kidney diseases, have not been reported in the public domain. There may be reasons for this lack of preclinical data. The mechanism of action of CNI is well established. Mouse models of SLE respond to the traditional CNI, especially in terms of proteinuria control [67,68]. In addition, mice are not a sensitive tool for a subtle drug safety analysis. In the clinical setting, most LN trials testing modulators of adaptive immunity failed to reach the primary endpoint that heavily relies on proteinuria control [69]. Indeed, an anti-proteinuric drug may be more likely to reach standard endpoints in LN trials, with or without a capacity to suppress autoimmunity, than a potent immune modulator because the processes underlying proteinuria only indirectly relate to the immune system [70]. Hence, immunotherapies acting outside of the kidney can control proteinuria only with some delay, while antiproteinuric drugs, including CNI, show a more rapid response [59]. Finally, the market potential of LN may exceed that of other autoimmune and proteinuric kidney diseases [3].

### 5.2. Clinical Efficacy Data of Voclosporin in Lupus Nephritis

Clinical efficacy of VOC in LN was tested in the phase 2 AURA-LV and the phase 3 AURORA-1 studies in 265 patients requiring treatment for a first diagnosis or relapse of biopsy-proven LN [53,71]. The AURA-LV study tested two oral doses of 23.7 mg VOC or 39.5 mg VOC or a placebo as an add-on to 2g/d mycophenolate mofetil (2 g/d), and rapidly tapered oral corticosteroids [71]. A total of 32.6% of the low-dose VOC group achieved a complete renal response at week 24, 27.3% of the high-dose VOC group, and 19.3% of the placebo group. The significantly greater CRR rate in the low-dose VOC group persisted for up to 48 weeks [71].

The AURORA-1 trial included SLE patients who had a kidney biopsy within 2 years showing proliferative LN class III or IV, membranous LN class V, or combinations of these [53]. Patients received either oral 23.7 mg VOC twice daily or a placebo as add-on to a background of mycophenolate mofetil (2 g/d) and rapidly tapered oral corticosteroids. The primary endpoint of a complete renal response at 52 weeks was defined as a composite of proteinuria of 0.5 or less, stable estimated glomerular filtration rate (eGFR) ≥60 mL/min/1.73 m^2^, or no GFR decline from baseline of >20%, no rescue medication, and no more than 10 mg prednisone equivalent per day [53]. The 357 patients were randomized in a 1:1 ratio. A total of 41% of VOC-treated patients reached a complete renal response at week 52 compared to 23% of the placebo group with an odds ratio of 2.65; 95% CI 1.64–4.27; *p* < 0.0001). The majority of patients reached proteinuria treatment targets as defined by the current EULAR/ERA recommendations [69,72]. These are similar to what has been proposed for lupus nephritis by the global KDIGO guidelines [73]. It is of note that a difference in proteinuria control between the two groups was evident as early as two weeks after the start of VOC, which is unique among the non-CNI drugs used in the LN sphere. Meanwhile 2 year follow-up analyses have been reported that document a persistent benefit of VOC-treated patients in terms of this endpoint [74]. It is of note that the parameters of systemic autoimmunity and extrarenal manifestations of SLE were hardly assessed in this trial [53]. Complement and anti-dsDNA levels were not much different between the groups further supporting the predominant antiproteinuric effect of VOC [53]. Thus, the two trials confirmed that the antiproteinuric effect of CNI in combination with MMF is potent enough to meet traditional trial endpoints in LN in patients from various world regions, validating earlier Chinese studies. Thus, VOC shares the immunosuppressive and antiproteinuric effects with the traditional CNI.

### 5.3. Clinical Safety Data of Voclosporin in Lupus Nephritis

Even novel immune modulators are still associated with adverse events, namely infectious complications [75]. The safety analysis of the AURA-LV trial revealed more serious adverse events in both VOC groups, and more deaths in the low-dose group compared to the placebo and high-dose VOC groups (11.2%, 1.1%, and 2.3%, respectively) [71]. Although these results raised some concerns, the safety analysis of the AURORA-1 trial displayed different results. Pneumonia occurred in 4% of VOC and 4% of placebo-treated patients [53]. Six patients died during the study and follow-up period, of which only one was treated with VOC. None of the fatal events were considered related to the study treatments. Similarly, in the AURORA 2 follow-up study, a total of 216 patients continued from the AURORA 1 trial, among which 116 were VOC treated while the rest were in the control group; the investigators found that the treatment with VOC did not increase chronic injury and helped in minimizing the renal histological changes [76]. These two follow-up data from different studies suggest that VOC is safer, and other side effects matched the known safety profile of CNI, such as occasional hypertension or a transient drop in GFR [53]. However, as judged by the estimated GFR, nephrotoxicity was not observed during the reported two years of follow up. GFR remained stable in both treatment groups [74]. Other adverse effects known from CsA or TAC such as gingival hyperplasia, diabetes or hyperlipidemia were not observed [53]. Thus, VOC seems to have a better safety profile than other CNI, especially regarding metabolic disturbances and long-term nephrotoxicity.

### 5.4. How to Integrate Voclosporin into the Treatment Landscape of Lupus Nephritis

**Figure 2:**
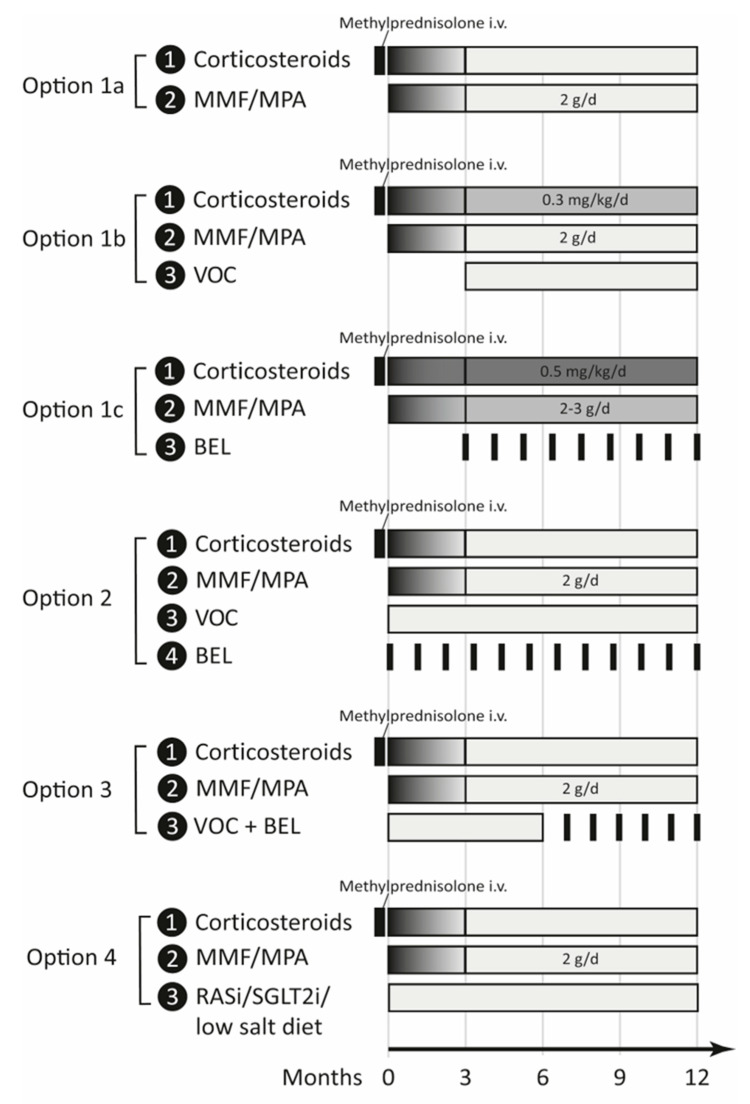
Four different options for the implementation of voclosporin into the treatment landscape of lupus nephritis. The different colors of grey indicate relative dosage. MMF = mycophenolate mofetil, MPA = mycophenolic acid, VOC = voclosporin, BEL = belimumab, RASi = renin-angiotensin system inhibitor, SGLT2i = sodium-glucose transporter-2 inhibitor.

Based on the results of the AURORA-1 trial, numerous countries approved VOC for the initial treatment of active LN [10]. This approval is very welcome in the community but raises numerous questions about how to implement VOC into the current treatment landscape. Meanwhile, Belimumab (BEL) had also been approved for the same indication based on the results of the BLISS-LN trial [77]. In contrast to VOC, BEL is well known to rheumatologists as BEL has been in use for the treatment on non-renal SLE since 2011 and has demonstrated good long-term efficacy on SLE activity and non-renal SLE manifestations at a comfortable safety profile [78]. As such data are lacking for VOC, the predominant advantage of VOC over BEL seems to be its capacity to control proteinuria much faster than BEL [53,77]. Rapid control of proteinuria might help to avoid irreversible kidney injury early in the disease course [79], but data supporting this concept are still pending. Indeed, the AURORA-1 trial included a subset of patients that underwent repeat biopsy, which would allow us to see if VOC-treated patients develop less chronic lesions. However, for the moment, a specific renoprotective effect of early proteinuria control remains speculative. Various concepts of how to implement VOC in the management of LN are possible (Figure 2).

#### 5.4.1. Option 1—Patient Selection

Rovin et al. started out with a pragmatic proposal to consider BEL and VOC as second line drug options only for those patients not adequately responding to standard-of-care within the first 3 months of treatment [80]. They proposed a threshold of 25% reduction in proteinuria as a marker of non-response [80] but without specifying whether this includes low-salt diet, a maximal tolerated dose of renin-angiotensin-system (RAS) inhibitors, and an inhibitor of sodium-glucose transporter-2 (SGLT2) or not, which all have substantial impact on proteinuria levels independent from the immunological SLE activity [70]. The choice for either BEL or VOC would consider an individual cost–benefit assessment as performed elsewhere [81]. Rovin et al. proposed a preference of BEL over VOC in patients on MMF with a residual proteinuria of <3 g/d [80]. This option acknowledges that a significant number of patients can reach a treatment response without any of the new drugs and that limited resource settings may not be able to afford the new drugs in all patients. However, as a limitation, a partial proteinuria response as a guidance factor is subject to numerous confounders of proteinuria as a criterion for treatment decisions in LN. For example, being overweight, having a salty diet, non-use of RAS inhibition, and the use of dihydropyridin therapy to control hypertension may explain an insufficient drop in proteinuria not necessarily requiring BEL or VOC add-on therapy but other interventions.

#### 5.4.2. Option 2—Combination Therapy

BEL and VOC both improve responder rates, but still a significant number of patients do not reach a complete renal response [53,77]. Thus, combining the B cell deactivator BEL and the T cell suppressor and podocyte protector VOC may have synergistic effects in controlling all aspects of LN. As of now, this concept has not been tested in a controlled study and not even case reports or series have been reported. Therefore, caution is warranted regarding possible drug interferences and toxicity profiles. However, as belimumab is not metabolized in the liver, the risk for interferences may be limited to a possible additive effect on host defense and infectious complications. Given the different sponsors of BEL and VOC, such a trial is unlikely to be conducted. As another limitation, costs for such a treatment regime would hardly be affordable in most regions of the world.

#### 5.4.3. Option 3—Sequential Therapy

The strength of VOC is rapid proteinuria control [53], which qualifies VOC for immediate use in the management of LN in cases where rapid proteinuria control would be associated with long-term benefits, e.g., by avoiding irreversible injury in this phase. By contrast, the mechanism of action of BEL is rather long-term by controlling the autoreactive lymphocytes clones that drive SLE activity and trigger flares of LN [82]. In this regard, one could imagine the sequential use of VOC and BEL or starting LN therapy with both of them and stopping VOC after 6 months when the effect of BEL kicks in. However, such a regimen would be costly and data to support a sequential therapy regimen are lacking so far.

#### 5.4.4. Option 4—Other Antiproteinuric Drugs

In health care settings with limited availability or reimbursement of VOC, CsA and TAC may appear as more affordable alternatives given they have the same mechanism of action [3]. However, the main advantages of VOC over these drugs are that it lacks the need for drug level monitoring and the much better safety profile. Nephrotoxicity especially limits the use of CsA on the long-term [5], as recently confirmed in the MENTOR trial where CsA was tested against Rituximab in primary membranous nephropathy [61]. Other antiproteinuric drugs that might replace VOC in this regard in the early phase of disease exist, e.g., RAS inhibitors or SGLT2 inhibitors, which reduce proteinuria by modulating glomerular hemodynamics and filtration pressure [83]. Such drugs lack any immunosuppressive effects, but it is currently unclear to what extent the long-term results of the AURORA-1 trial relate to the immunosuppressive effects of VOC or to their antiproteinuric effects at the filtration barrier [53]. However, in this case, VOC would be preferentially given in a sequential therapy approach, these drugs may to some extent substitute for the rapid antiproteinuric effect of VOC and even contribute to a renoprotective effect by antagonizing the non-immune mechanisms of LN progression [84,85]. This might be a possibility in settings with limited resources for costly drugs. However, RAS/SGLT2 blockades may make more sense, when used in combination with BEL to suppress SLE activity and LN relapses by targeting B cell activity.

### 5.5. Cost of Voclosporin Treatment

The results from clinical trials related to VOC treatment in LN showed VOC’s clinical effects on LN. Though the results are more in favor of VOC, the cost-effectiveness of VOC will be a challenging task in the future as there is uncertainty around the cost-effectiveness of VOC. According to one study, the cost-effectiveness of VOC is approximately USD 150,000 per quality-adjusted life years (QALYs) in the United States [86]. Notably, the other emerging drugs, such as BEL, have approximately USD 95,000 per QALY, suggesting that BEL was more cost effective [86]. Moreover, VOC costs GBP 1000 per 180-pack of 7.9 mg soft capsules, while standard therapies such as MMF cost around GBP 6.23 per 50-pack of 500 mg tablets (https://www.nice.org.uk/guidance/ta882/resources/voclosporin-with-mycophenolate-mofetil-for-treating-lupus-nephritis-pdf-82613730259141, Accessed on 11 January 2023). Costs will certainly have an effect on the use and implementation of VOC into clinical practice especially in limited resource settings.

## 6. Conclusions

As CsA and TAC remain associated with significant toxicities, the introduction of VOC into clinical care is an important advancement of treatment options for LN and possibly other autoimmune and proteinuric kidney diseases. VOC has a different chemistry, pharmacology, and safety profile compared with CsA and TAC, namely, VOC seems to lack nephrotoxicity. These aspects are an important asset when considering CNI for chronic treatment of LN [3]. However, the problem of interference with other drug classes that can cause additional toxicities in the context of certain co-medications remains. The available evidence supports rapid and robust proteinuria control, which is different from other immunosuppressants in use and in development for LN. Whether rapid proteinuria control prevents irreversible injury better than standard of care is not yet known but is possible. This potential advantage would imply immediate and early use of VOC in LN but may not necessarily argue for continued VOC therapy. Data on the potential of VOC to control SLE and non-renal SLE manifestations are scarce, while these are robust for alternative drug options such as BEL. How VOC will find its place in the changing treatment landscape of LN is not yet clear.

## Figures and Tables

**Table 1:** The dosage, clinical indications, and adverse effects of calcineurin inhibitors.

Drugs	Clinical Use	Contraindications/ Adverse Effects
Cyclosporine	Solid organ transplantation (liver, kidney, and heart), rheumatoid arthritis, psoriasis, amyotrophic lateral sclerosis, nephrotic syndrome, graft vs. host disease (GVHD), refractory posterior uveitis, and Behcet disease. Off-label: Allergic conjunctivitis, autoimmune hepatitis, keratoconjunctivitis, Langerhans cells histiocytosis, Duchenne muscular dystrophy, ocular graft vs. host disease, ulcerative colitis, pure red cell aplasia, Henoch Schönlein purpura nephritis, proteinuric forms of glomerulonephritis and podocytopathies.	Contraindications: Amphotericin B, neomycin, atorvastatin, cidofovir, elbasvir/grazoprevir, flibanserin, lomitapide, mifepristone, tacrolimus, life vaccines, etc. Adverse effects: Dyslipidemia, hyperkalemia, gynecomastia, hypertension, arrhythmia, decrease in eGFR and creatinine clearance, convulsions, bleeding gums, GIT upset, infectious complications.
Tacrolimus	Solid organ transplantation (liver, kidney, and heart. Off-label: Crohn’s disease, Graft-versus-host disease (GVHD), Myasthenia gravis, and Rheumatoid arthritis, Ulcerative colitis, proteinuric forms of glomerulonephritis and podocytopathies.	Contraindications: some antifungal agents, polyoxyl 60 hydrogenated castor oil (HCO-60), and derivatives. Adverse effects: Nephrotoxicity, neurotoxicity, post-transplant diabetes mellitus, hypertension, dyslipidemia, angina pectoris, cardiac arrhythmias, urinary tract infections, cosmetic and electrolyte disturbances, infectious complications.
Voclosporin	Active lupus nephritis. Off-label: Plaque psoriasis, prevention of organ rejection after transplantation, uveitis, arthritis, and Crohn’s disease.	Contraindications: Phenylalanine, flunisolide, bortezomib, cladribine, and in patients with renal and hepatic impairments. Adverse effects: Reduced eGFR, increased blood pressure, diarrhea, headache, anemia, cough, UTI, upper abdominal pain, dyspepsia, alopecia, renal dysfunction, abdominal pain, mouth ulceration, nausea, tremor, acute kidney injury and decreased appetite, infectious complications.

**Table 2:** Comparison of major adverse effects of cyclosporine and tacrolimus with voclosporin.

Adverse Effects	Cyclosporin	Tacrolimus	Voclosporin
Hypertension	++	+	+
Nephrotoxicity	++	++	+
Decrease in eGFR	+	+	+
Arrhythmia/cardiovascular risk	+	+	−
Anemia	+	+	+
Neurotoxicity/convulsions	+	++	+
Gastrointestinal upset	+	++	+
Gum hyperplasia	++	−	+
Dyslipidemia	++	+	−
Gynecomastia	+	−	−
Alopecia	−	++	+
Post-transplant diabetes mellitus	+	++	−
Urinary tract infection		+	+
Hyperkalemia	++	+	+
Cosmetic and electrolyte disturbances	++	+	−
Hyperuricemia	++	+	−
Hirsutism/hypertrichosis	++	−	−

++: more pronounced side effects, +: less pronounced side effects, −: no side effects.

## Data Availability

Not applicable.
